# Overweight and obesity knowledge prior to pregnancy: a survey study

**DOI:** 10.1186/1471-2393-11-96

**Published:** 2011-11-21

**Authors:** Marloes Dekker Nitert, Katie F Foxcroft, Karin Lust, Narelle Fagermo, Debbie A Lawlor, Michael O'Callaghan, H David Mcintyre, Leonie K Callaway

**Affiliations:** 1 School of Medicine, Royal Brisbane Clinical School, The University of Queensland, Brisbane, Australia; 2 Department of Internal Medicine Research Unit, Royal Brisbane and Women's Hospital, Brisbane, Australia; 3 Department of Maternity Services and Internal Medicine & Aged Care, Royal Brisbane and Women's Hospital, Brisbane, Australia; 4 Department of Social Medicine, University of Bristol, Bristol, UK; 5 Mater Children's Hospital, Brisbane, Australia; 6 Departments of Endocrinology and Obstetric Medicine, Mater Health Services, Brisbane, Australia; 7 Centre for Diabetes and Endocrine Research, The University of Queensland, Bribane, Australia; 8 Department of Internal Medicine, Royal Brisbane and Women's Hospital, Brisbane, Australia

## Abstract

**Background:**

Overweight and obesity are associated with increased risk for pregnancy complications. Knowledge about increased risks in overweight and obese women could contribute to successful prevention strategies and the aim of this study is to assess current levels of knowledge in a pregnant population.

**Methods:**

Cross sectional survey of 412 consecutive unselected women in early pregnancy in Brisbane, Australia: 255 public women attending their first antenatal clinic visit and 157 women at private maternal fetal medicine clinics undergoing a routine ultrasound evaluation prior to 20 weeks gestation. The cohort was stratified according to pre pregnancy BMI (< 25.0 or ≥ 25.0). The main outcome measure was knowledge regarding the risks of overweight and obesity in pregnancy.

**Results:**

Over 75% of respondents identified that obese women have an increased risk of overall complications, including gestational diabetes and hypertensive disorders of pregnancy compared to women of normal weight. More than 60% of women asserted that obesity would increase the risk of caesarean section and less than half identified an increased risk of adverse neonatal outcomes. Women were less likely to know about neonatal complications (19.7% did not know about the effect of obesity on these) than maternal complications (7.4%). Knowledge was similar amongst women recruited at the public hospital and those recruited whilst attending for an ultrasound scan at a private clinic. For most areas they were also similar between women of lower and higher BMI, but women with BMI < 25.0 were less likely to know that obesity was associated with increased rate of Caesarean section than those with higher BMI (16.8% versus 4.5%, P < 0.001). Higher educational status was associated with more knowledge of the risks of overweight and obesity in pregnancy.

**Conclusions:**

Many women correctly identify that overweight and obesity increases the overall risk of complications of pregnancy and childbirth. The increased risks of maternal complications associated with being obese are better known than the increased risk of neonatal complications. Maternal education status is a main determinant of the extent of knowledge and this should be considered when designing education campaigns.

## Background

In line with the age and gender adjusted general population prevalence [1], approximately one third of pregnant women in Australia are overweight (BMI 25-29.9) or obese (BMI > 30) [2,3]. These rates are similar to the rates in other developed and developing countries [4]. Arguably, overweight and obesity are currently among the most common risk factors for adverse pregnancy outcomes [5]. Table 1 provides an overview of the quoted prevalence and odds ratios for a number of pregnancy and neonatal complications for obese women compared to women of normal weight, derived from a detailed literature review in this area. These complications include gestational diabetes, hypertensive disorders, caesarean section, thromboembolism, perinatal infections and in the neonate high birth weight or macrosomia, higher rates of intensive care nursery admission, congenital anomalies, preterm delivery, stillbirth and perinatal death [1,6,1,1,1,15-40]. Obesity in pregnancy is therefore associated with greater direct costs of $ 2387 (CI: $1799-$3109; *P *< 0.0001) per pregnancy [41].

**Table 1:** Prevalence and odds ratios for pregnancy and birth complications

Pregnancy and Birth Complications	Prevalence in normal weight women	Prevalence in obese women	Range of Odds ratios -obese women	Range of Odds ratios -Class II and or III obesity
Gestational diabetes	1.2-4.1% ^14, 17, 18, 54 ^	3.5-9.5% ^14, 15, 17, 18, 23, 54^	2.6-5.2 ^15, 16, 18, 20, 23, 54^	4-7.4 ^14, 17, 18^
Hypertensive disorders of pregnancy	0.7-4.8% ^14, 17, 18^	1.4-13.5% ^14, 15, 17, 18 ^	2.1-5.2 ^13, 14-16, 18, 20^	3.2-4.9 ^10, 14, 17, 18^
Caesarean section	7.7-22.3% ^10, 14, 17^	10.4-36.2% ^14, 15, 17^	1.7-2.9 ^15, 16, 17, 20^	2.5-3.0 ^14, 16, 17^
Premature birth < 37 weeks	5.4-19.6% ^14, 16, 17, 18^	6.4-30.7% ^12, 14, 15, 17, 18^	0.9-1.6* ^15, 18, 20, 38^	1.5-1.85 ^17, 18^
Special care nursery admission	4.3-9.3% ^17^	6-33.2% ^17^	1.2-1.3 ^16^	1.4-3.4 ^16^
Congenital abnormality	1.2-4.5% ^16, 22, 23, 36^	2.2-5.5% ^22-24, 29, 31-33, 36^	1.1-2.6* ^22-24, 36^	1.4-3.4 ^14, 22, 29^

The odds ratios represent a range of published unadjusted odds ratios, confidence intervals are not included. Class II obesity, BMI between 35.0 and 39.9 kg/m^2^; Class III obesity, BMI ≥ 40.0 kg/m^2^. * Published odds ratios have 95% confidence intervals crossing 1.0, implying that the relationships are not statistically significant.

Increasing women's knowledge of the short and long-term risks of obesity to both their own and their offspring's health is likely to be an important first step in preventing obesity in pregnancy. Indeed recommendations to improve preconception care emphasize the need to ensure that women of childbearing age understand factors that increase the risks of childbearing, including obesity [42]. Our study was designed to ascertain whether or not women in the general pregnant population were aware of the increased risks associated with obesity in pregnancy. Furthermore, we investigated whether or not the pre pregnancy BMI was associated with differences in risk perception for complications in obese women.

## Methods

We developed a questionnaire and surveyed 412 consecutive unselected women in early pregnancy as previously reported [43]. These women were either attending a public antenatal "first visit" clinic (n = 255), or undergoing a routine private ultrasound evaluation prior to 20 weeks gestation (n = 157)[44]. 61.9% of study participants were cared for in the public sector, similar to previously published proportions from Queensland [45]. Pre pregnancy BMI was available for 368 women. Women completed the survey independently while waiting for appointments. A trained research midwife was present at all times, to assist if participants required clarification regarding any component of the survey. The response rate for the questions varied between 96 and 100%. Permission for this study was obtained from the Royal Brisbane and Women's Hospital Health Research and Ethics Committee.

### Data collection

Participants were asked to rate their perception of the risk of a pre-specified list of seven maternal and neonatal complications for women who were 'very underweight', 'normal weight' and 'very obese'. For each complication and with each weight status women were asked to indicate level of risk using a 5 point Likert scale (very low risk, low risk, average risk, high risk, very high risk, in addition to a "don't know" option). The specific questions used are shown in Additional file 1.

Participants were also asked "If a very obese woman was able to lose weight before pregnancy, how do you think this would affect her risk of pregnancy and birth complications?" The same seven factors were rated on a 5 point Likert scale using the following descriptors: She would be at much lower risk, She would be at lower risk, There would be no change in risk, She would be at higher risk, She would be at much higher risk (see Additional file 1).

### Definition of knowledge about the risks of being obese prior to pregnancy

We assessed the way in which women rated risk for each complication for a normal and very obese woman. For the purposes of more detailed analysis, we evaluated women's broad knowledge about the risks of pregnancy and birth complications associated with being very obese. To be categorized as having broad knowledge about the risks of being very obese, women needed to rate the overall risk of complications as high or very high, and had to identify that weight loss prior to pregnancy is associated with a lower or much lower overall risk of complications.

### Factors associated with knowledge about the risks of being obese

A number of demographic and obstetric history questions were included in the questionnaire. We explored the univariable and independent (of all other factors considered) associations of characteristics that we a priori thought were likely to be associated with knowledge and that might be useful in determining which groups of women should be specifically targeted to increase knowledge. The factors considered in these analyses were: maternal age (categorized as < 25 years, 25-35 years, > 35 years), parity (categorized as nulliparous or multiparous), smoking during current pregnancy (yes versus no), personal income (categorized as > 40 000 or ≤ 40 000 AUD per year), obstetric care (classified as public or private), pregnancy planning (categorized as planned or unplanned), highest educational status (classified as < Year 12, completed Year 12 or completed a tertiary qualification), body mass index (BMI) prior to pregnancy derived from self-report of pre pregnancy weight and height (categorized as < 25 kg/m^2 ^or **≥**25 kg/m^2 ^), periconceptual folate supplementation (yes versus no), attendance at a pre pregnancy planning visit with a doctor (yes versus no), weight loss attempts prior to current pregnancy (yes versus no), previous history of pregnancy-induced hypertension (yes versus no), of gestational diabetes (yes versus no) and of neonatal morbidity or mortality (including low birth weight baby, preterm baby, baby with a birth defect, death of baby within 1 month, baby requiring special or intensive care nursery).

### Statistical analysis

Differences between women with a pre pregnancy BMI < 25.0 or ≥25.0 were analysed by two-sided Χ^2 ^tests. P < 0.05 was considered statistically significant. Logistic regression was used to assess the relationship between each explanatory variable and "knowledge" of the risks of being very obese prior to pregnancy. Continuous variables (maternal age, BMI) were explored both as continuous and categorical variables, to ensure that this did not have an important effect on any of the multiple logistic regression models. Variables with several categories (parity, personal income) were explored using the original multiple categories and the dichotomized variable presented here in the results, to ensure that this did not substantially alter any of the odds ratios presented here. Multivariable logistic regression was used to further investigate some of the positive associations that we found. All analyses were performed with the statistical software package STATA v11.0.

## Results

**Figure 1:**
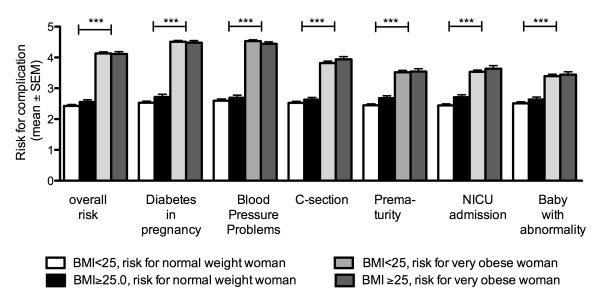
**Risks for maternal and infant complications for a normal weight woman or a very obese woman respectively as assessed by pregnant women with a pre pregnancy BMI < 25.0 (white box and light grey box respectively) or BMI ≥ 25.0 (black box and dark grey box respectively) on a five point Likert scale**. Results are expressed as mean ± SD. N = 354 for women with BMI < 25.0 and 111 for women with BMI ≥ 25.0. ***, P < 0.001 between the risks for a normal weight woman and an obese woman. There were no statistically significant differences between the assessments of women with a pre pregnancy BMI < or ≥ 25.0 kg/m^2^.

The baseline characteristics of the participating women are presented in Table 2. There was no difference in the baseline characteristics between women with a pre pregnancy BMI of < 25.0 and those with ≥ 25.0, except for BMI itself. Participants were asked to rate risk for a normal weight and a very obese woman for a variety of pregnancy and birth outcomes. These results are stratified by BMI < 25.0 or ≥ 25.0 and presented in Figure 1 and Additional file 2. There were no statistically significant differences in the responses from the women in the two BMI categories; all rated the risk for adverse pregnancy and birth outcomes higher for a very obese woman. In general, women were more confident of the effect of obesity on maternal than neonatal outcomes with 9.0-16.8% and 20.7-22.3% responding "Don't Know" in the BMI < 25.0 and 4.5-13.6% and 14.5-19.2% in the BMI ≥ 25.0 group for the different maternal and neonatal outcomes respectively. A majority of women rated the risks for a very obese woman of overall complications (74.6% vs. 71.6%), gestational diabetes (87.8% vs. 86.5%), blood pressure problems (88.2% vs. 88.3%) or caesarean section (53.6% vs. 50.7%) as high to very high, whereas the risk of preterm delivery (62.8% vs. 60.9%), admission to special nursery care (63.9% vs. 59.1%) and congenital anomalies (58.0% vs. 62.7%) were rated as average to high in the BMI < 25.0 vs. BMI ≥ 25.0 groups respectively.

**Table 2:** Participant demographic characteristics.

	BMI < 25.0	BMI ≥ 25.0	P-value
N	257	111	
Age	31.6 ± 4.9	31.4 ± 5.9	0.75
Nulliparous (%)	48.8	41.7	0.40
Gestation (weeks)	19.1 ± 6.0	20.0 ± 6.6	0.18
Pregnancy planned (%)	65.3	66.1	0.88
Prepregnancy health check (%)	47.5	43.5	0.47
Periconception folic acid supplements (%)	41.1	45.2	0.46
Tertiary degree (%)	61.5	62.6	0.84
Public hospital patient (%)	57.0	67.0	0.07
Born in Australia (%)	74.2	78.3	0.40
Smoking (%)	21.1	20.0	0.80
Prepregnancy BMI (kg/m^2^)	21.1 ± 2.2	30.9 ± 5.7	**< 0.0001**

Students t-test was used to compare between the groups for continuous variables and Chi^2 ^for categorical variables.

In Table 3 data is presented regarding how participants rated the risk of a very obese woman in comparison to a woman of normal weight, again stratified according to pre pregnancy BMI. The majority of the respondents were aware of the increased risk of overall complications, gestational diabetes and hypertensive disorders in obese women whereas a smaller proportion identified higher risks for caesarean section, adverse neonatal outcomes and especially congenital anomalies. There were no significant differences in the responses of women with a pre pregnancy BMI < 25.0 compared to those with a BMI ≥ 25.0.

**Table 3:** Participant rated risk of pregnancy and childbirth complications for women with a BMI < 25.0 or ≥ 25.0

	Don't know (n(%))		Very obese woman at lower risk than normal weight woman (n(%))		Very obese woman at the same risk as normal weight woman (n(%))		Very obese woman at increased risk compared to normal weigh woman (n(%))	
	BMI < 25	BMI ≥ 25	BMI < 25	BMI ≥ 25	BMI < 25	BMI ≥ 25	BMI < 25	BMI ≥ 25
Overall risk of complications	27 (10.6)	14 (12.8)	1 (0.4)	0 (0)	23 (9.1)	112 (11.0)	203 (79.9)	83 (76.2)
Gestational diabetes	28 (11.1)	12 (10.9)	1 (0.4)	0 (0)	13 (5.2)	11 (10.0)	210 (83.3)	87 (79.1)
Hypertension in pregnancy	27 (10.7)	11 (9.9)	0 (0)	0 (0)	15 (6.0)	11 (9.9)	210 (83.3)	89 (80.2)
Caesarean section	50 (19.8)	19 (17.1)	2 (0.8)	1 (0.9)	58 (23.0)	27 (24.3)	142 (56.3)	64 (57.7)
Prematurity	57 (22.5)	19 (17.3)	5 (2.0)	4 (3.6)	63 (24.9)	44 (40.0)**	128 (50.6)	43 (39.1)*
Special Care Nursery Admission	55 (21.8)	20 (18.2)	0 (0)	4 (3.6)	77 (30.4)	40 (36.4)	121 (47.8)	46 (41.8)
Congenital abnormality	62 (24.6)	23 (20.9)	1 (0.4)	2 (1.8)	93 (36.9)	44 (40.0)	96 (38.1)	41 (37.3)

Total number of participants answering varies slightly (n = 252 to 254 for women with BMI < 25, n = 109 to 111 for women with BMI ≥ 25). *p = 0.04

**p = 0.004. All neonatal outcomes and C-section are rated as "dont' know"more frequently than maternal outcomes in both obese and non obese women p < 0.001

The majority of respondents thought that weight loss prior to pregnancy would lower the risk of all pregnancy and birth complications independent of their own pre pregnancy BMI (Table 4).

**Table 4:** Responses regarding change in risk if an obese woman were to lose weight prior to pregnancy

	Lower or much lower risk (n(%))		No change in risk (n(%))		Higher or much higher risk (n(%))	
	BMI < 25	BMI ≥ 25	BMI < 25	BMI ≥ 25	BMI < 25	BMI ≥ 25
Overall risk of complications	197 (80.4)	83 (77.6)	19 (7.8)	13 (12.1)	29 (11.8)	11 (10.3)
Gestational diabetes	191 (78.3)	78 (72.9)	24 (9.8)	17 (15.9)	29 (11.9)	12 (11.2)
Hypertension in pregnancy	185 (75.8)	77 (72.6)	29 (11.9)	17 (16.0)	30 (12.3)	12 (11.2)
Caesarean section	150 (63.0)	65 (60.7)	62 (25.6)	31 (29.0)	30 (12.4)	11 (10.3)
Prematurity	146 (60.3)	54 (55.7)	67 (27.7)	34 (35.1)	29 (12.0)	9 (9.3)
Special Care Nursery Admission	149 (61.6)	62 (57.9)	66 (27.3)	35 (32.7)	27 (11.2)	10 (9.4)
Congenital abnormality	127 (52.7)	56 (52.3)	86 (35.7)	41 (38.3)	28 (11.6)	10 (9.4)

The total number of participants answering each question varied from n = 241-245 for women with BMI < 25.0 and n = 97-107 for women with BMI ≥ 25.0

Two hundred and thirty-five women (57% of the total cohort independent of pre pregnancy BMI) were categorized as knowing about the risks of being obese on pregnancy, birth and neonatal outcomes. Table 5 provides information about a number of variables that we hypothesized might be related to knowledge about the risks of overweight and obesity. Educational status was consistently associated with knowledge of overweight and obesity prior to pregnancy. Women who were cared for in the private sector were more likely to be categorized as having a broad knowledge of the risks of overweight and obesity. These women were also more likely to have attended a preconception visit (98 of 157 women with private care (62.4%) vs. 122 of 255 women with public care (47.8%), P < 0.01). We adjusted this analysis for maternal educational status, and found that increased maternal educational status fully explained the difference in knowledge between women cared for in the private and public sector.

**Table 5:** Association between demographic variables and broad knowledge of obesity-related risk for pregnancy complications and outcomes.

	Total	Broad know-ledge about absolute risks		Unadjusted Analysis	
		n	%	OR	95% CI
**Maternal Age**					
< 25	46	21	45.6	1	
25-35	234	140	59.8	1.77	0.94, 3.35
> 35 yrs	132	73	55.3	1.47	0.75, 2.89
**Educational status**					
Did not complete secondary school	108	49	45.4	1	
Completed secondary school	160	89	55.6	1.51	0.92, 2.47
Tertiary degree	144	96	66.7	**2.41**	**1.44, 4.02**
**Parity at birth**					
Nulliparous	179	110	61.5	1	
Multiparous	233	124	53.2	0.71	0.48, 1.06
**Pregnancy planning**					
Unplanned	82	75	51.4	1	
Planned	266	159	59.8	1.19	0.97, 1.45
**Obstetric care**					
Private	155	100	63.7	1	
Public	257	134	52.6	**0.63**	**0.42, 0.95**
**Smoking status during pregnancy**					
Did not smoke in pregnancy	321	188	58.6	1	
Smoked at all in pregnancy	91	46	50.6	0.72	0.45, 1.15
**Family Income**					
> $ 40 000/yr	182	109	59.9	1	
≤ $40 000/yr	190	112	58.9	0.96	0.63, 1.45
**BMI Pre pregnancy**					
< 25	265	160	60.4	1	
≥ 25.0	115	65	56.5	1.23	0.83, 1.83
**Periconception folic acid supplementation**					
No	180	97	53.9	1	
Yes	232	139	59.1	0.96	0.65, 1.42
**Pre Pregnancy Health Check**					
No	192	110	57.3	1	
Yes	220	124	56.4	0.85	0.55, 1.33
**Weight loss attempts prior to pregnancy**					
No	267	148	55.4	1	
Yes	134	79	59	1.07	0.87, 1.33
**Previous hypertensive disorders of pregnancy**					
No	193	105	54.4	1	
Yes	42	20	47.2	0.76	0.39, 1.49
**Pre gestational or gestational diabetes**					
No	185	222	57.5	1	
Yes	48	12	46.1	0.63	0.28, 1.40
**Previous neonatal morbidity or mortality**					
No	386	97	52.4	1	
Yes	26	27	56.2	1.17	0.61, 2.21

## Discussion

57% of the women in this study knew that being very obese prior to pregnancy increased the overall risk of pregnancy and birth complications, and that weight loss prior to pregnancy in an obese woman would reduce the overall risk of complications. The responses did not differ between normal weight and overweight or obese women.

The majority of women correctly identified the impact of overweight and obesity on maternal complications including diabetes and hypertensive disorders developing in pregnancy. The impact of pre pregnancy weight on caesarean section rates and neonatal outcomes was less well known (Figure 1 and Additional file 1). This is perhaps not surprising, given that relative risks are lower than for maternal adverse outcomes (Table 1). In addition, the increased risk of preterm delivery and congenital abnormalities is not consistently reported in the literature, until women are extremely obese (Class II and III obesity) although recent meta-analyses have indicated increased risks for both overweight and obese women [38,40]. Given that a healthy baby is a highly valued outcome of pregnancy [46], increasing women's knowledge about the impact on overweight and obesity on neonatal problems such as birth defects might encourage women to actively attempt to lose weight prior to pregnancy. A meta-analysis of Leventhal's common-sense models as a theoretical basis for intervention programs identified moderate to strong relationships between knowledge of disease, coping behaviors and outcomes [47]. Tailored diet and exercise interventions for at-risk individuals have been shown to be effective in improving outcomes in type 2 diabetes in a number of studies [48-50]. Therefore a program that will encompass an increase in knowledge of the risks of obesity for maternal and neonatal pregnancy outcomes with tailored easily implementable lifestyle interventions may improve pregnancy outcome for obese women.

Tertiary degree qualification was associated with knowledge about the risks of overweight and obesity. Maternal educational status also fully explained the difference we found in knowledge of the risks of being obese between women cared for in the private and public sectors and between women who did or did not smoke during pregnancy. Educational status is an important predictor of birth outcomes [51], and is associated with better knowledge of other preconception health issues such as periconceptual folate supplementation [5]. Our data would suggest that to improve knowledge regarding the risks of obesity, targeting public health messages at those with lower levels of education would be important.

This study identifies the pre pregnancy health check as an excellent opportunity for improving education of women regarding the risks of obesity prior to pregnancy. Slightly more than half of all women attended a doctor for a pre pregnancy health check. It is important that women have their BMI determined at their pre pregnancy health check, are advised about the risks associated with pre pregnancy overweight and obesity, and where appropriate are provided with support to lose weight [52]. However, this study also showed that education levels are associated with the level of knowledge and preconception visits to health care professionals, and efforts to increase knowledge about the risks associated with obesity during pregnancy in women with lower education levels should include additional measures besides information during preconception visits.

### Strengths and Limitations

This study provides information on risk perception relating to the influence of being overweight and obese on pregnancy and birth complications in a relatively large unselected cohort of pregnant women cared for in the private and public sectors. Given the dearth of previous information in this area, we believe that our data will provide useful information to help develop public health interventions for reducing optimizing preconception weight mas well as providing a baseline against which to measure changes in knowledge after future interventions.

We were concerned that this cohort might have been particularly skewed towards well educated women. Women in our cohort had only slightly higher rates of tertiary education (34.9% vs. 28.8%), and similar rates of secondary school non completion (26.2% vs. 27.4%) in comparison to national Australian data [1,17,20,53,54], hence this should not be a major source of bias in this study. It is possible that the responses in this survey might have been positively influenced by local media coverage regarding the problems of overweight and obesity which occurred at around the time of questionnaire administration and it would be useful to repeat this survey again in this population and also in other populations.

It would be worthwhile to conduct a similar survey in health professionals, to assess their understanding of the risks associated with being overweight and obese prior to conception. A detailed knowledge in this group, of the adverse health consequences associated with elevated BMI on pregnancy would be associated with opportunities to address weight loss preconception. This would be especially amongst general practitioners, who generally would provide preconception check ups and could target women requiring weight loss prior to conception.

All the outcomes that we examined are associated with obesity. One of the limitations of this study is that we did not include a false outcome to test whether participants simply assumed all adverse outcomes would be more common in obese women (reflecting the relatively widespread portrayal as obesity as a major contributor to general ill-health). However, the relative risk of each outcome comparing obese to non-obese women does vary in the published literature (Table 1) and knowledge of the effect of obesity on outcomes with a lower relative risk was lower in this survey, indicating that the results may reflect real knowledge.

## Conclusions

This study provides evidence that many women correctly identify that overweight and obesity increases the overall risk of complications of pregnancy and childbirth and that this was independent of the woman's own BMI. There remains scope for improvement in women's knowledge about obesity as a risk factor for pregnancy, birth and neonatal complications. Less well educated women are less likely to know about the risks of overweight and obesity in pregnancy, and so future public health campaigns need to ensure that these women are specifically considered.

## Competing interests

The authors declare that they have no competing interests.

## Authors' contributions

MN performed data analysis and drafted the manuscript. KF/NF/KL performed data collection. DL/MC participated in data analysis. HM/LC conceived and designed the study, participated in data analysis and helped draft the manuscript. All authors have critically reviewed the manuscript and approved the final manuscript.

## Pre-publication history

The pre-publication history for this paper can be accessed here:

http://www.biomedcentral.com/1471-2393/11/96/prepub

## Supplementary Material

Additional file 1 **Appendix 1**. The appendix contains the list of questions posed to the participants in the survey regarding the knowledge of the effects of overweight and obesity in pregnancy. Click here for file

Additional file 2 **Participant response (%) for risk of complications for women with BMI < and BMI ≥ 25**. This file contains the tabular results for the data presented graphically in Figure 1. Click here for file
