# Supplementary material for: Overweight and obesity knowledge prior to pregnancy: a survey study
Source: BMC Pregnancy Childbirth. 2011 Nov 21;11:96. doi: 10.1186/1471-2393-11-96 (PMC3240826; doi:10.1186/1471-2393-11-96)
Supplement: Additional file 1 — Appendix 1. The appendix contains the list of questions posed to the participants in the survey regarding the knowledge of the effects of overweight and obesity in pregnancy. [file 1471-2393-11-96-S1.DOC]

Appendix 1:

**Overweight and obesity are becoming very common in Australia. These questions are to help us understand how much pregnant women know about this issue. You are welcome to guess the answers. *Please do not worry if you do not know the answers*.**

What would you think are the risks of pregnancy and birth complications in a woman of normal weight?

|  | | Very Low Risk | Low Risk | Average risk | High risk | Very high risk | Don’t Know |
| --- | --- | --- | --- | --- | --- | --- | --- |
| **Q 124**. | Overall risk of complications | 1 | 2 | 3 | 4 | 5 | 6 |
| **Q 125.** | Diabetes in Pregnancy | 1 | 2 | 3 | 4 | 5 | 6 |
| **Q 126.** | Blood pressure problems in pregnancy | 1 | 2 | 3 | 4 | 5 | 6 |
| **Q 127**. | Caesarean section | 1 | 2 | 3 | 4 | 5 | 6 |
| **Q 128.** | Baby being born prematurely | 1 | 2 | 3 | 4 | 5 | 6 |
| **Q 129**. | Baby needing admission to the special care nursery | 1 | 2 | 3 | 4 | 5 | 6 |
| **Q 130.** | Baby being born with an abnormality | 1 | 2 | 3 | 4 | 5 | 6 |

**What would you think are the risks of pregnancy and birth complications in a woman who is very obese**?

|  | | Very Low Risk | Low Risk | Average risk | High risk | Very high risk | Don’t Know |
| --- | --- | --- | --- | --- | --- | --- | --- |
| **Q 131**. | Overall risk of complications | 1 | 2 | 3 | 4 | 5 | 6 |
| **Q 132.** | Diabetes in Pregnancy | 1 | 2 | 3 | 4 | 5 | 6 |
| **Q 133**. | Blood pressure problems in pregnancy | 1 | 2 | 3 | 4 | 5 | 6 |
| **Q 134.** | Caesarean section | 1 | 2 | 3 | 4 | 5 | 6 |
| **Q 135.** | Baby being born prematurely | 1 | 2 | 3 | 4 | 5 | 6 |
| **Q 136.** | Baby needing admission to the special care nursery | 1 | 2 | 3 | 4 | 5 | 6 |
| **Q 137**. | Baby being born with an abnormality | 1 | 2 | 3 | 4 | 5 | 6 |

**If a very obese woman was able to lose weight prior to pregnancy, how do you think this would affect her risk of problems during pregnancy and birth complications?**

|  | | She would be at **much lower** risk | She would be at **lower** risk | There would be **no change** in risk | She would be at **higher** risk | She would be at **much higher** risk |
| --- | --- | --- | --- | --- | --- | --- |
| **Q 138**. | Overall risk of complications | 1 | 2 | 3 | 4 | 5 |
| **Q 139**. | Diabetes in Pregnancy | 1 | 2 | 3 | 4 | 5 |
| **Q 140.** | Blood pressure problems in pregnancy | 1 | 2 | 3 | 4 | 5 |
| **Q 141.** | Caesarean section | 1 | 2 | 3 | 4 | 5 |
| **Q 142**. | Baby being born prematurely | 1 | 2 | 3 | 4 | 5 |
| **Q 143.** | Baby needing admission to the special care nursery | 1 | 2 | 3 | 4 | 5 |
| **Q 144.** | Baby being born with an abnormality | 1 | 2 | 3 | 4 | 5 |
