# Supplementary material for: Overweight and obesity knowledge prior to pregnancy: a survey study
Source: BMC Pregnancy Childbirth. 2011 Nov 21;11:96. doi: 10.1186/1471-2393-11-96 (PMC3240826; doi:10.1186/1471-2393-11-96)
Supplement: Additional file 2 — Participant response (%) for risk of complications for women with BMI < and BMI ≥ 25. This file contains the tabular results for the data presented graphically in Figure 1. [file 1471-2393-11-96-S2.DOC]

| **Supplementary Table 1: Participant response (%) for risk of complications for women with BMI<25 and BMI≥25.** | | | | | | | | | | | | | | | | | |
| --- | --- | --- | --- | --- | --- | --- | --- | --- | --- | --- | --- | --- | --- | --- | --- | --- | --- |
|  | Very Low Risk | |  | Low Risk | |  | Average Risk | |  | High Risk | |  | Very High Risk | |  | Don’t Know | |
| BMI | <25 | ≥25 |  | <25 | ≥25 |  | <25 | ≥25 |  | <25 | ≥25 |  | <25 | ≥25 |  | <25 | ≥25 |
| **Overall risk of complications** |  |  |  |  |  |  |  |  |  |  |  |  |  |  |  |  |  |
| Normal weight woman | 12.8 | 12.6 |  | 30.4 | 16.2 |  | 52.5 | 61.3 |  | 0 | 0.9 |  | 0.4 | 0 |  | 3.9 | 9.0 |
| Very overweight woman | 0.4 | 0 |  | 1.2 | 0.9 |  | 13.7 | 15.6 |  | 45.7 | 44.1 |  | 28.9 | 27.5 |  | 10.1 | 11.9 |
| **Diabetes in pregnancy** |  |  |  |  |  |  |  |  |  |  |  |  |  |  |  |  |  |
| Normal weight woman | 10.1 | 9.0 |  | 30.1 | 20.7 |  | 48.4 | 9.6 |  | 3.9 | 6.3 |  | 1.2 | 3.6 |  | 6.3 | 10.8 |
| Very overweight woman | 0.8 | 0 |  | 0.4 | 0.9 |  | 2.0 | 60.3 |  | 36.2 | 33.3 |  | 51.6 | 53.2 |  | 9.0 | 6.3 |
| **Blood pressure problems in pregnancy** |  |  |  |  |  |  |  |  |  |  |  |  |  |  |  |  |  |
| Normal weight woman | 8.1 | 8.1 |  | 30.9 | 23.4 |  | 47.7 | 51.4 |  | 4.3 | 4.5 |  | 2.7 | 3.6 |  | 6.3 | 9.0 |
| Very overweight woman | 0.4 | 0 |  | 0.4 | 0.9 |  | 2.0 | 6.3 |  | 35.8 | 37.8 |  | 52.4 | 50.5 |  | 9.0 | 4.5 |
| **Ceasarean section** |  |  |  |  |  |  |  |  |  |  |  |  |  |  |  |  |  |
| Normal weight woman | 9.0 | 9.0 |  | 27.0 | 18.9 |  | 52.7 | 58.6 |  | 0.8 | 3.6 |  | 0.8 | 0 |  | 9.7 | 9.9 |
| Very overweight woman | 1.6 | 0 |  | 1.6 | 3.6 |  | 26.4 | 26.1 |  | 34.3 | 28.8 |  | 19.3 | 27.9 |  | 16.8 | 13.6 |
| **Baby born prematurely** |  |  |  |  |  |  |  |  |  |  |  |  |  |  |  |  |  |
| Normal weight woman | 10.5 | 7.2 |  | 34.0 | 20.7 |  | 46.5 | 57.7 |  | 2.3 | 2.7 |  | 0.4 | 1.8 |  | 6.3 | 9.9 |
| Very overweight woman | 1.2 | 0 |  | 3.9 | 7.3 |  | 35.7 | 41.8 |  | 27.1 | 19.1 |  | 9.8 | 17.3 |  | 22.3 | 14.5 |
| **Baby admitted to special care nursery** |  |  |  |  |  |  |  |  |  |  |  |  |  |  |  |  |  |
| Normal weight woman | 11.3 | 8.1 |  | 32.0 | 17.1 |  | 44.9 | 60.4 |  | 2.8 | 1.8 |  | 0.4 | 2.7 |  | 8.6 | 9.9 |
| Very overweight woman | 0.8 | 0.9 |  | 2.7 | 3.6 |  | 38.8 | 39.1 |  | 25.1 | 20.0 |  | 9.8 | 19.1 |  | 20.7 | 17.3 |
| **Baby born with abnormality** |  |  |  |  |  |  |  |  |  |  |  |  |  |  |  |  |  |
| Normal weight woman | 9.0 | 9.0 |  | 31.6 | 20.7 |  | 46.5 | 55.0 |  | 0 | 2.7 |  | 2.7 | 1.8 |  | 10.2 | 10.8 |
| Very overweight woman | 1.2 | 0.9 |  | 7.1 | 4.5 |  | 38.4 | 47.3 |  | 19.6 | 15.4 |  | 9.8 | 12.7 |  | 23.9 | 19.2 |
